# Experience with Ether Narcosis up to the Present Time

**Published:** 1894-07

**Authors:** 


EXPERIENCE WITH ETHER NARCOSIS UP TO THE PRESENT TIME—Dr. P. Tschmarke (*Deutsche Med. Wochenschr.*, 1894; Vol. XX., p. 79.) Having had a death after the use of chloroform, and incited by the recommendations of ether narcosis from other countries, the use of the latter was commenced in the surgical department of the hospital Moabit in Berlin. The quick method of etherization was always employed, and about one ounce of ether was at once poured on the sponge, followed by a few drachms more as necessary. The result can be summarized as follows:

1. Ether narcosis appears to be less dangerous than chloroform narcosis, and at the same time is quite as easy and simple in its application, and can be used for all ages.

2. Ether narcosis is indicated in heart diseases, since ether increases the heart pressure.

3. Ether narcosis should not be employed—

(*a*) In operations of the face or its immediate neighborhood, especially when thermo-cautery is used.

(*b*) When bronchitis or any other lung trouble is present.

(*c*) When it is necessary to obtain complete relaxation of the muscles quickly.

4. In these cases chloroform is to be used as formerly.

5. Chloroform by no means to be discarded, as each narcotic has its undoubted advantages.—*Ex.*

